# Institutional Needs

**Published:** 1915-10-30

**Authors:** 


### Institutional Needs.

### "PERMUTIT" WATER SOFTENER.

(United Water Softeners, Ltd., Imperial House, Kingsway, London, W.C.)

One of the results of the present war is the vastly increased amount of attention now being devoted to water purification and softening by hospitals and institutions. The high prices of coal have made it imperative to minimise the waste incidental to the feeding of boilers and hot-water systems with hard water, while the great advance in the price of soap and other washing materials has compelled attention to the question of softening the water used in the laundry and the baths. There is overwhelming proof that large savings can be effected by softening the water used for washing, and that this is generally realised is proved by the general adoption of water softeners in hospital laundries. The main objections to softeners on the part of hospital authorities have been the troubles incidental to the use of lime and the disposal of sludge; and in this connection it is worthy of note that the recently perfected "Permutit" process entirely obviates the necessity of lime treatment or sludge formation, whilst at the same time producing a perfectly softened water, which has lost none of its original palatability. We are informed by the United Water Softeners, Ltd., that fourteen hospitals and institutions have been recently equipped with the "Permutit" process, with gratifying results.

